# Realization of Self-Adaptive Higher Teaching Management Based Upon Expression and Speech Multimodal Emotion Recognition

**DOI:** 10.3389/fpsyg.2022.857924

**Published:** 2022-03-28

**Authors:** Huihui Zhou, Zheng Liu

**Affiliations:** ^1^School of Education, University of Perpetual Help System DALTA, Las Piñas, Philippines; ^2^School of Hunanities and Communications, ZheJiang GongShang University, Hangzhou, China; ^3^School of Journalism, Fudan University, Shanghai, China

**Keywords:** emotion recognition, higher education, facial expressions, teaching management, self-adaptive

## Abstract

In the process of communication between people, everyone will have emotions, and different emotions will have different effects on communication. With the help of external performance information accompanied by emotional expression, such as emotional speech signals or facial expressions, people can easily communicate with each other and understand each other. Emotion recognition is an important network of affective computers and research centers for signal processing, pattern detection, artificial intelligence, and human-computer interaction. Emotions convey important information in human communication and communication. Since the end of the last century, people have started the research on emotion recognition, especially how to correctly judge the emotion type has invested a lot of time and energy. In this paper, multi-modal emotion recognition is introduced to recognize facial expressions and speech, and conduct research on adaptive higher education management. Language and expression are the most direct ways for people to express their emotions. After obtaining the framework of the dual-modal emotion recognition system, the BOW model is used to identify the characteristic movement of local areas or key points. The recognition rates of emotion recognition for 1,000 audios of anger, disgust, fear, happiness, sadness and surprise are: 97.3, 83.75, 64.87, 89.87, 84.12, and 86.68%, respectively.

## Introduction

Emotion recognition plays an important role in people's social interactions. The research goal of recognizing emotions in human-computer interaction is to allow computers to achieve more natural and friendly interactions by “understanding” and ultimately recognizing human emotional states. Preliminary research on emotion recognition has focused on identifying emotional features. Throughout the research process, it became clear that there is a limit to a single pathway, and that the emotional properties of different channels are somewhat complementary. Therefore, multimodal emotion recognition has become a research hotspot. The purpose of teaching management is to optimize the use of teaching resources and maximize teaching benefits. The role of teaching management technology is to maximize management capabilities.

Multimodal emotion recognition has become a hot research area in the field of emotion recognition. Expression and language are the two most direct ways to express human emotions. Research on these two kinds of emotion recognition can enrich the field of emotion recognition. The theoretical results of this research will help to deeply explore the theoretical algorithms related to multimodal emotion detection, and promote the development and innovation of emotion detection technology, which is of great significance. On the one hand, teaching management is the rational arrangement of teachers, students, classrooms, and teaching equipment. On the other hand, it is the improvement of various teaching management systems, as well as the communication and cooperation between various management departments. In this process, the efficiency and accuracy of information exchange are extremely important.

The innovation of this paper is to construct multimodal emotion recognition of expressions and speech. In this paper, two normalization methods are used to obtain the recognition results of facial expressions and speech, respectively. Finally, the six emotional audios are recognized, respectively, to obtain their respective recognition rates. In this paper, a control group and an experimental group were set up to conduct positive emotional group counseling, and additional tests were conducted on the emotional state of college students before and after counseling and whether they used learning strategies.

## Related Work

Regarding emotion recognition, related scientists have done the following research. Jenke et al. reviews feature extraction methods for EEG emotion recognition based on 33 studies. He conducted comparative experiments on these features using machine learning techniques for feature selection on self-recorded datasets. He gives results on the performance of different feature selection methods, the use of selected feature types, and electrode location selection. The multivariate method selects features slightly better than the univariate method. He found that advanced feature extraction techniques have advantages over commonly used spectral power bands. The results also indicated a preference for parietal and central parietal locations (Jenke et al., 2017). Emotion is a key element of user-generated video. However, due to the complex and unstructured nature of user-generated content and the sparseness of video frames expressing emotion, it is difficult to understand the emotions conveyed in such videos. Xu et al. first proposed a technique to transfer knowledge from heterogeneous external sources, including image and text data, to facilitate three related tasks of understanding video emotion: emotion recognition, emotion attribution, and emotion-oriented (Xu et al., 2018). One of the challenges in virtual environments is that it is difficult for users to interact with these increasingly complex systems. Ultimately, giving machines the ability to sense user emotions will make interactions more intuitive and reliable. Menezes investigated features extracted from EEG signals to model affective states based on Russell's Circumplex model. The survey he presents aims to provide a basis for future work in modeling user influence to enhance interactive experiences in virtual environments. Wankhade and Doye aims to identify human emotional states or influences through EEG signals by employing advanced feature and classifier models. In the first stage of the recognition process, 2,501 (EMCD) and wavelet transforms are utilized to represent low-dimensional and descriptive EEG signals. Through EMCD, EEG redundancy can be ignored and important information can be extracted. And it demonstrates the superiority of the proposed work in identifying emotions more accurately (Wankhade and Doye, 2020). Zheng proposed a correlation analysis method for simultaneous EEG channel selection and emotion recognition. GSCCA is a group-sparse extension of traditional canonical correlation analysis methods to model linear correlations between emotion EEG category label vectors and corresponding EEG feature vectors. For EEG emotion recognition, he uses common frequency features to describe EEG signals. Finally, extensive experiments are conducted on EEG-based emotion recognition, and the experimental results show that the proposed GSCCA method will outperform ECG-based emotion recognition methods (Zheng, 2017). Albornoz and Milone proposes a novel system to model each language independently, preserving cultural attributes. In the second stage, the concept of emotional universality is used to map and predict emotions in never-before-seen languages. His widely tested features and classifiers for similar tasks are used to set the baseline. He developed a novel ensemble classifier to handle multiple languages and tested it on never-before-seen languages. Results show that the proposed model improves baseline accuracy, while its modular design allows the incorporation of new languages without training the entire system (Albornoz and Milone, 2017). Automatic recognition of spontaneous facial expressions is a major challenge in the field of affective computing. Happy et al. proposes and builds a new Facial Expression Database. In the Experiments, Emotions Were Evoked in Participants by Using emotional videos, while self-ratings of each experienced emotion were collected. Facial expression clips were carefully annotated by four trained decoders and further validated by self-reports of the nature and mood of the stimuli used. He performed extensive analysis of the database using several machine learning algorithms and provided the results for future reference (Happy et al., 2017). Huang et al. proposes that stimuli are based on clipped subsets corresponding to four specific regions (happy, neutral, sad, and fearful) of the emotional space of valence arousal. The results show that the accuracy rates of the two multimodal fusion detections are 81.25 and 82.75%, respectively, which are higher than those of facial expression (74.38%) or EEG detection (66.88%). The combination of facial expression and EEG information for emotion recognition makes up for their shortcomings as a single source of information (Huang et al., 2017). These methods provide some references for research, but due to the short time and small sample size of the relevant research, this research has not been recognized by the public.

## The Realization Method of Self-Adaptive Higher Education Management

### Emotion Recognition

The computer analyzes and processes the signals collected from the sensor to obtain the emotional state of the other party (person), which is called emotion recognition. From a physiological psychology point of view, emotion is a complex state of an organism that involves both experience and physiological responses, as well as behavior. Its components include at least three factors: emotional experience, emotional performance and emotional physiology. There are currently two methods for emotion recognition, one is to detect physiological signals such as respiration, heart rate and body temperature. The other is to detect emotional behaviors such as facial feature expression recognition, speech emotion recognition and gesture recognition.

Emotion is a human feeling and intention that can be expressed explicitly or indirectly through a specific vehicle. Emotion recognition includes several categories: mainly the perception of emotional behavior (facial expressions, voice, posture, etc.) and physiological patterns (galvanic skin response, heart rate, breathing, body temperature, etc.). While these categories can express emotions independently, in communication and interpersonal interactions, people often express many universal emotions simultaneously. In real life, people need to consider many things before making a decision (Bedford et al., 2017).

Language is one of the main forms in people's daily communication process, including the expression and transformation of emotions in the process of information transmission. The characteristics of speech signals are not static. Speech is a signal that develops over time and contains very rich information. When determining the emotional points of a language, the short-term prosodic features, spectral signatures, and other statistical notations of speech are first considered. As shown in Table 1, the speech feature parameters under various emotions are shown. The numbers in the table indicate the level, the larger the number, the higher the level.

**Table 1:** Changes of speech feature parameters under various emotions.

	**Happy**	**Sad**	**Fear**	**Disgust**	**Angry**
Pitch frequency	6	3	6	1	6
Pitch range	6	3	6	3	6
Voice intensity	5	2	4	2	5
Voice rate	5	2	6	1	3

The video stream is obtained by combining multiple frames of images. In the recognition based on the video stream, it is necessary to obtain the facial expression area image of the key frame from the video image. Video stream expression recognition: It has a high dependence on image preprocessing; it focuses on the research on the overall process from image preprocessing to expression classification; the classification algorithm generally uses static expression recognition directly. Expression recognition of static images: generally do not need to consider image pre-processing; focus on improving the efficiency of expression recognition; research algorithms have strong robustness to occlusion and interference.

Facial feature extraction is the process of processing and refining the original image information. The effect of feature extraction is to preserve their features as much as possible, which is useful for sensory analysis. According to the different methods of face imaging, it can be divided into three categories: deriving general features, deriving partial features, and deriving hybrid features (Schuller, 2018).

Emotion recognition classification is the last step in the emotion recognition system. Currently, the commonly used emotion recognition classifiers are roughly as follows: artificial neural network, K-nearest neighbor classifier, hidden Markov model, and support vector machine.

For an image, the meaning it is trying to convey can be viewed from a global or local perspective. For the printed image, the general information after feature separation is relatively complete, but the general features of the output statistical analysis cannot be fully represented, so the recognition rate may be relatively low. The advantages of this method are that it is simple and efficient, but the amount of data is large, the computer cost is high, and the final level of feature recognition is low.

EEG signals can directly reflect the activities of the central nervous system, so it is the most effective and direct source of information to study human perception and emotional expression. EEG signal is a random signal of non-stationary time series, its frequency range is 0.5–50 Hz, and the signal is very weak. Neurophysiological findings suggest that the characteristics of EEG frequency range are closely related to its performance (Lee and Yong, 2017).

Assuming that the pixel value at (*m, n*) in the grayscale image is *f* (*m, n*), there are:


(1) $$\begin{array}{l} f\;(m,n)=a\times R\;(m,n)+b\times G\;(m,n)+c\times B\;(m,n) \end{array}$$

(2) $$\begin{array}{l} k_{i}\;(m)=\begin{array}{c} 1\\ 2\pi \alpha_{win} \end{array}\exp\;(-\begin{array}{c} 1\\ 2 \end{array}\begin{array}{c} {\;(m-m_{i})}^{2}\\ \alpha_{win}^{2} \end{array})\;\beta \;(\begin{array}{c} m\\ a\alpha \end{array}) \end{array}$$

(3) $$\begin{array}{l} k_{j}\;(m)=\begin{array}{c} 1\\ 2\pi \alpha_{win} \end{array}\exp\;(-\begin{array}{c} 1\\ 2 \end{array}\begin{array}{c} {\;(n-n_{i})}^{2}\\ \alpha_{win}^{2} \end{array})\;\beta \;(\begin{array}{c} n\\ a\alpha \end{array}) \end{array}$$

The discretized gray histogram equalization Formula is:


(4) $$\begin{array}{l} \beta_{k}=U\;(\alpha_{k})=\overset{k}{\underset{n=0}{\sum }}L_{\alpha }\;(\alpha_{n})=\overset{k}{\underset{n=0}{\sum }}\begin{array}{c} s_{n}\\ M \end{array} \end{array}$$

(5) $$\begin{array}{l} l\;(t,i,j)=\;(h_{i}h_{j}\cdot J_{i})\;(B+a\alpha \left[\begin{array}{c} m_{i}\\ m_{j} \end{array}\right]) \end{array}$$

*k*-discrete grayscale

*M*-total pixels of the image

The brightness value of each pixel in the linear correction method can be expressed as:


(6) $$\begin{array}{l} P\;(m,n)=\frac{298R\;(m,n)+586G\;(m,n)+112B\;(m,n)}{1000} \end{array}$$

(7) $$\begin{array}{l} J_{i}\;(x)=\beta_{ang}\;(∠J\;(x)-\theta_{t})J\;(x) \end{array}$$

Then the corresponding probability density function is:


(8) $$\begin{array}{l} H_{k}=\overset{k}{\underset{\alpha =1}{\sum }}d\;(s_{\alpha }) \end{array}$$

(9) $$\begin{array}{l} G\;(F_{M}^{l},F_{N}^{l})=\overset{j}{\underset{i=1}{\sum }}\min\;(F_{M}^{l}\;(i),F_{N}^{l}\;(i)) \end{array}$$

*s*_α_-The gray value of the pixel point

The calculation of the brightness value can be calculated by the following Formula:


(10) $$\begin{array}{l} P_{1}\;(m,n)=\begin{array}{l} F-V\\ \text{ln }N-\ln\;M \end{array}\left[\ln\;P\;(m,n)-\ln\;M\right]+V \end{array}$$

(11) $$\begin{array}{r} c\;(n)=\{1,\;0\leq n\leq N-1\\ 0,\;others \end{array}$$

*P*_1_ (*m, n*)-is the adaptive brightness value

*M*-the maximum value of the gray value of the original image

*N*-the minimum value of the gray value of the original image

*F*-The maximum gray value of the image after adaptive calculation

*V*-The minimum gray value of the image after adaptive calculation


(12) $$\begin{array}{l} P^{\prime }\;(m,n)=\{\begin{array}{l} 0,P\;(m,n)<U\\ 255\frac{\ln\;P\;(m,n)-\ln\;U}{\ln\;V-\ln\;U},U<P\;(m,n)<V\\ 255,P\;(m,n)>V \end{array} \end{array}$$

(13) $$\begin{array}{l} c\;(n)=\{\begin{array}{l} 0.5\;(1-\cos\;(2\pi n/\;(N-1))),0\leq n\leq N-1\\ 0,others \end{array} \end{array}$$

*U, V*-the brightness value of the pixel

Compute the probabilities of feature vectors relative to various emotions:


(14) $$\begin{array}{l} Tb=\overset{n}{\underset{x=1}{\sum }}wifi\;(m) \end{array}$$

(15) $$\begin{array}{l} c\;(n)=\{\begin{array}{l} 0.54-0.46\cos\left[2\pi n/\;(N-1)\right],0\leq n\leq N-1\\ 0,others \end{array} \end{array}$$

*m*-d-dimensional speech emotion feature vector

*fi*-Gaussian distribution function


(16) $$\begin{array}{l} f\;(m)=\begin{array}{l} 1\\ 2\pi^{\pi }2{\left|\sum x\right|}^{1}2\exp\left[-\frac{1}{2}{\;(m-\alpha x)}^{Q}\sum_{x}^{-1}\;(m-\alpha x)\right] \end{array} \end{array}$$

The pre-processed speech signal is:


(17) $$\begin{array}{l} R\;(n)=M\;(n)-\omega *M\;(n-1) \end{array}$$

(18) $$\begin{array}{l} Mel\;(f)=2459\times \mathrm{lg}\begin{array}{l} \;(1+\frac{f}{600}) \end{array} \end{array}$$

ω-Pre-emphasis factor

*M* (*n*)-Sampling rate speech signal

*f*-the frequency of the original input signal

Feature points are described as follows:


(19) $$\begin{array}{l} l\;(t,i,j)=a\alpha \int g\alpha_{win}\;(x-R)\beta_{ang}\;(∠J\;(x)-\theta_{1}) \end{array}$$

(20) $$\begin{array}{l} B_{n}=\overset{j}{\underset{i=1}{\sum }}\log X\;(i)\cos\left[\pi \;(i-0.5)\;n/j\right],n=1,2,\ldots ,L \end{array}$$

*i*-Number of filters

*R*-Sampling step size

*aα*-bin size

**Figure 1:**
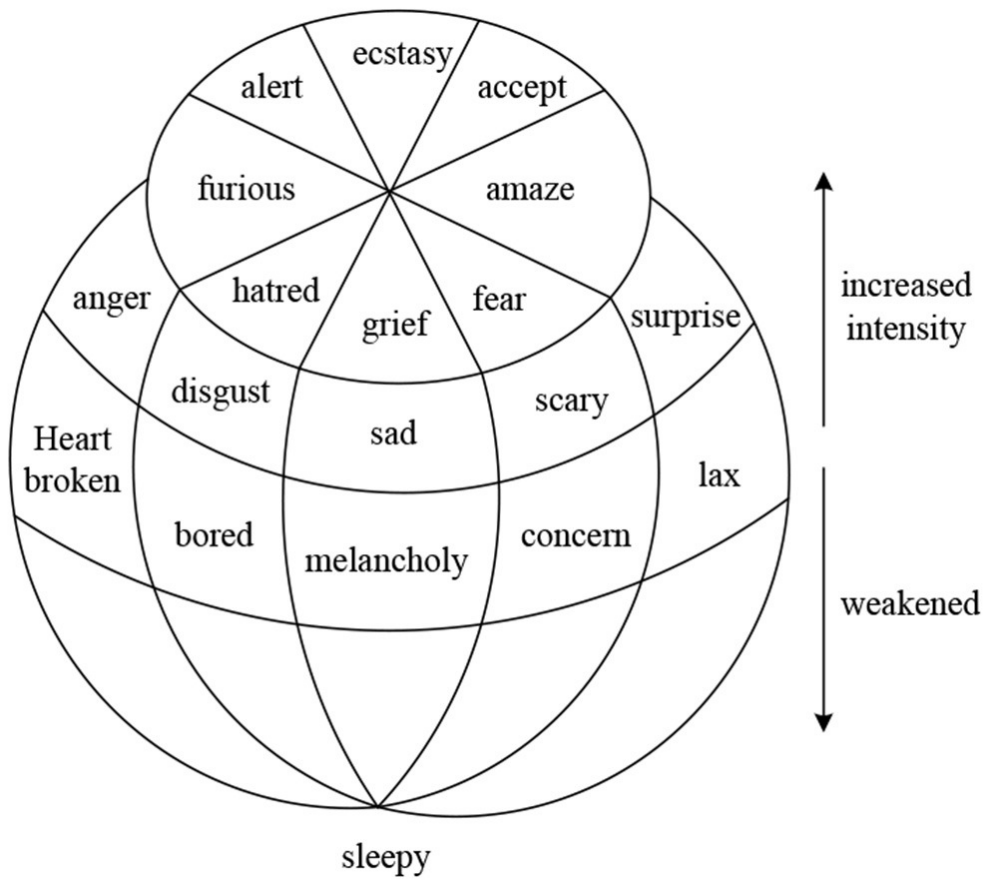
Basic emotion schematic.

As shown in Figure 1, the left side is a flat circular structure. The eight basic emotions are evenly distributed in the eight regions, and their combination can evoke all emotions (Thammasan et al., 2017). When the affective intensity factor is added and expanded vertically, the affective model can be considered three-dimensional.

In fact, there are certain objective motivations for emotion recognition and emotion communication between people. Division of labor and cooperation is the most effective way for human beings to improve social productivity. In order to better divide labor and cooperate, on the one hand, people must show their value relationship to others in a timely and accurate manner through a certain “emotional expression.” On the other hand, it is necessary to timely and accurately understand and grasp the value relationship of the other party through a certain “emotion recognition” method, so as to analyze and judge the value relationship between each other on this basis, and then make correct behavioral decisions. In a word, the objective essence or objective motivation of emotion recognition is that people understand and master the value relationship of each other. Because of the different types of interests that exist between people, the emotions displayed by the other party are sometimes completely accurate, sometimes exaggerated and disguised, and sometimes the exact opposite. At this time, people need to constantly adjust and revise the objective value content of each other's emotional expressions, so that their own emotional recognition has higher timeliness, accuracy, and integrity.

### Higher Education Management

The curriculum plan is an important document of the school, which stipulates the requirements for the quality of education and training, and is the basic basis for organizing the educational process and Formulating educational goals. The curriculum should not only adhere to the laws of education and maintain a certain stability, but also adapt and change over time with the development of society, economy and technology. Once procedures are defined, they must be carefully organized and implemented.

Teaching operation management mainly includes the following aspects: formulation of curriculum syllabus, organization and management of classroom teaching links, daily teaching management, organization and management of practical teaching links, student status management, and teacher teaching workload management.

According to the teaching plan, the management of educational functions is the most important and decisive management of educational activities, and educational management is the main body of the management department. The main goal is to coordinate the whole school, coordinate from top to bottom, strictly abide by the teaching standards and various systems, maintain the stability of teaching functions, and ensure the quality of teaching (Muhammad and Alhamid, 2017). The management of training and teaching operation mainly includes the following aspects:

1. Curriculum development. The curriculum is in line with the core curriculum requirements put forward by the Ministry of Education, and the curriculum is Formulated in accordance with the principles and rules of the school, relevant teacher training is organized, and students are recognized and approved for graduation. The school syllabus responsible for implementing the curriculum content includes the educational objectives of the curriculum, the basic requirements of the curriculum content, the requirements for practical learning, the distribution of the curriculum and the necessary explanations. Each class must have its own syllabus, and each teacher must strictly abide by the syllabus.
2. The organization and management of classroom teaching. Classroom education is a basic form of teaching. The responsibilities of each middle school and each teaching and research department are: to select teachers with high academic level, rich teaching experience and good teaching influence as backbone teachers. Carefully prepare all disciplines, training sessions and introduce learning systems in the workplace. Organize teachers to read the syllabus carefully, organize writing or self-monitoring, and conduct self-evaluation on teaching quality. Organize teachers to learn teaching methods, protect heuristic learning, and ensure students have new ways of thinking about learning. Actively use modern educational technologies such as computer teaching and multimedia teaching to expand the field of educational informatization and improve the quality of teaching.
3. Management courses in daily life. The syllabus, timetable and examination timetable for this semester's courses should be developed and strictly followed to ensure curriculum consistency across the school. In the implementation of these three important forms and documents, there should be control systems and verification methods, and the implementation results should be recorded. During the implementation process, continuously understand the training information, strictly monitor the training process and the approval of program changes, and discover problems or accidents in the implementation process in a timely manner.

The main purpose of learning management is to ensure and improve the quality of learning. In order to achieve the best learning effect, it is necessary to create a good educational environment through scientific evaluation, qualitative analysis and seamless information feedback network (Torres-Valencia et al., 2017).

Do a good job in the quality management of the whole process. It includes quality management of the registration process; step-by-step process quality management and implementation of curriculum development; quality management of the educational process and quality of all aspects of the educational process. Provide adequate and modern library materials, information technology, e-learning, equipment, gymnasium, multi-purpose classrooms, and improve the quality of teacher services. The application of scientific examination management, the establishment of scientific examination procedures and working systems, the strict control of the examination process, the analysis of necessary questions and examination documents, and the record of examination work and teaching should be done well (García-Casal et al., 2018).

Teaching evaluation is an important means of macro-control of teaching. The evaluation of school teaching generally includes: evaluation of the overall teaching work of the school and secondary colleges, evaluation of majors, courses and various teaching infrastructure, evaluation of teachers' teaching quality,and student learning quality, etc. To carry out the evaluation of teaching work, clarify the goals, establish a scientific evaluation index system, lay a solid foundation, highlight the key points, and adhere to the principle of “promoting construction through evaluation and focusing on construction.”

Teaching evaluation needs a certain organizational form to complete. Schools and secondary colleges can set up teaching evaluation leading groups, and can also assign corresponding responsibilities to teaching committees and other organizations. At the same time, it insists on regular and institutionalized evaluation of teaching work. It takes the goal and content of teaching evaluation as the main content of daily teaching construction and management, realizes the combination of teaching evaluation and daily teaching management, and does not engage in formalism. The evaluation of teaching work should be combined with the school's incentive mechanism and restraint mechanism. To mobilize the enthusiasm of teachers and cadres through evaluation, so as to enhance the cohesion of teachers, students and staff.

**Figure 2:**
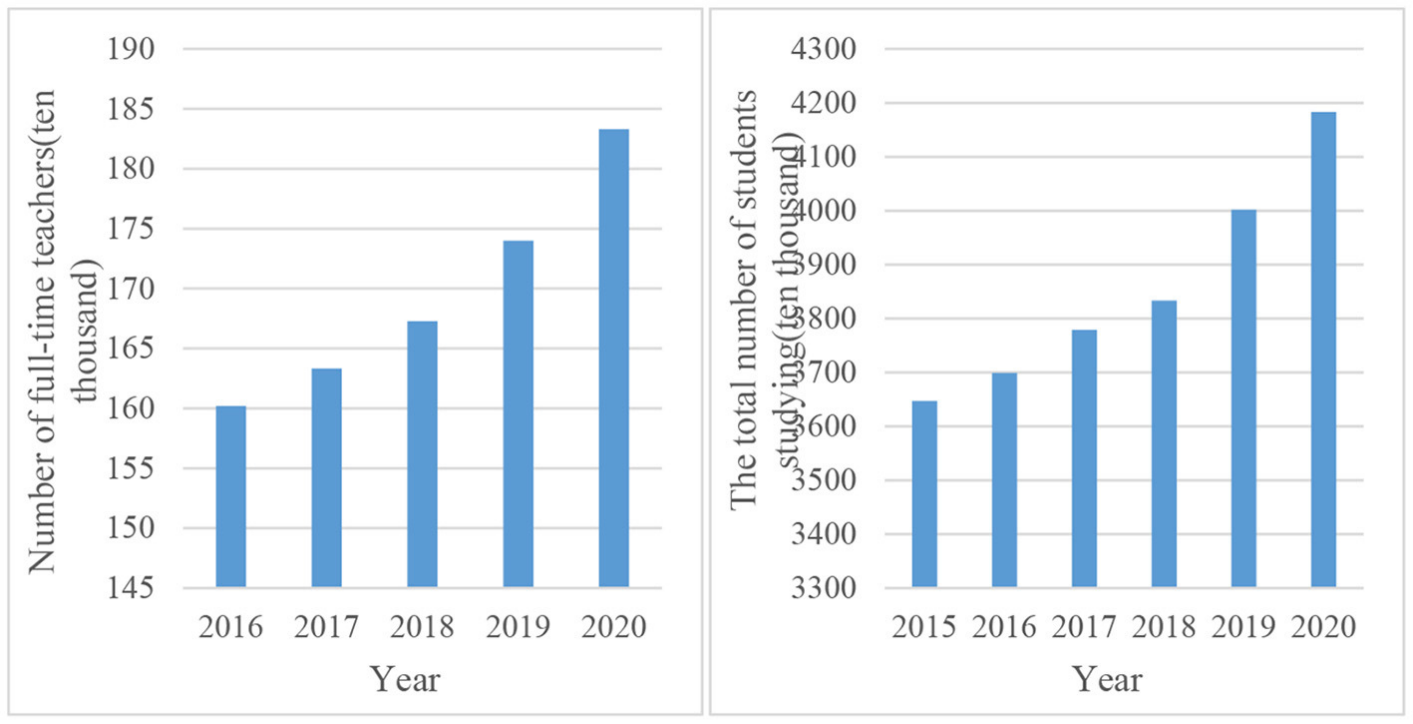
The number of full-time teachers in higher education and the total number of students in higher education.

Figure 2 shows the number of full-time teachers in China's higher education and the total scale of higher education in recent years.

## Experiments to Realize Self-Adaptive Higher Education Management

**Figure 3:**
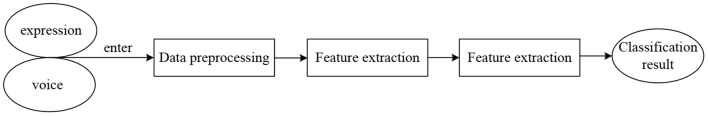
Framework of a dual-modal emotion recognition system based on facial expressions and speech.

Multimodal emotion recognition has been an important network for pattern detection, and through their research, researchers enable computers to recognize people's emotional states. People can express their emotions in different ways, but psychological research shows that language and facial expressions are the most direct way to express people's feelings and the most effective way to determine emotional state (Nakisa et al., 2018). As shown in Figure 3, the framework of the dual-modal emotion recognition system based on expression and speech is shown.

First of all, the input of emotional data in the first step needs to select and establish the emotional database used. The next step is to select the appropriate preprocessing method according to the data characteristics of different databases and the characteristics of different modal data. The third step, feature extraction, includes facial expression feature extraction and speech feature extraction. There are various methods of feature extraction, and different methods have different effects on different data. Finally, emotion recognition is mainly the selection and application of classifiers. At present, common methods include support vector machines, hidden Markov models, neural networks, and so on.

Emotional speech database is the premise of emotional speech recognition research, and its quality directly affects the reliability of the final result of emotion recognition. According to the different levels of emotional properties of emotional materials, the methods of emotional language library created by researchers can be divided into three types: natural speech, simulated speech and induced speech (Yan et al., 2018). As shown in Table 2, it is a representative speech database. This paper selects the Chinese speech database.

**Table 2:** Representative speech database.

**Language**	**Naturalness**	**Emotion type**	**Number of people**	**Number of statements**	**Multimedia information**
German	Simulation	Angry, irritable, annoying, scared, happy, sad, neutral	Five women, five men	535	Audio
Danish	Simulation	Angry, happy, neutral, sad, surprised	Two women, two men	419	Audio
English	Simulation and induction	Fearful, neutral	Three women, four men	1,185	Audio
German and English	Nature	Angry, happy, neutral, helpless, pensive, surprised	Forty-seven women and thirty-two men	2,774	Audio and video
Chinese	Simulation	Angry, scared, happy, sad, surprised, neutral	Two women, two men	7,200	Audio

**Figure 4:**
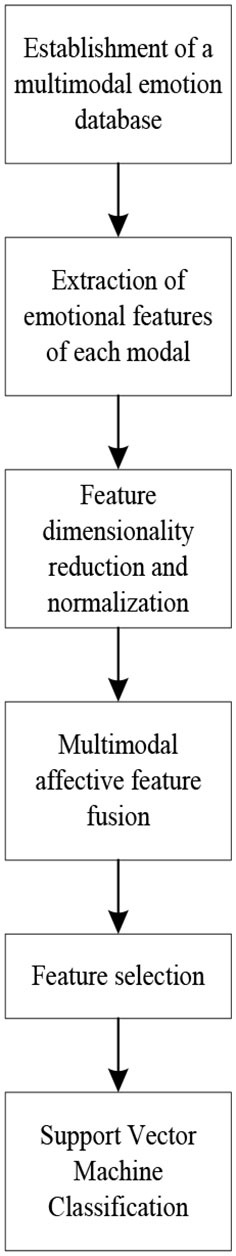
The basic process of multimodal emotion recognition.

As shown in Figure 4, the complete facial expression image is composed of partial image regions such as eyes, nose, and lips. Taking these local regions as the vocabulary that constitutes the image, the BOW model provides a higher-level description of the overall expression through the image blocks of local key parts. In this way, we describe a facial expression image as an unordered collection of features of some local regions or keypoints. The specific steps of BOW model generation include local feature extraction, codebook generation and image histogram representation (Kempnich et al., 2017).

1. Local feature extraction. A local attribute is distinguished in the image, forming a subdirectory of attribute descriptions. Attribute extraction methods usually include random extraction, dense sampling and point attribute extraction. It is better to keep the features invariant with changes in image transitions, brightness, and closure, and to retain rich information of varying qualities and widely varying.
2. The generated codebook adopts some aggregation methods to add the received attribute identifiers to K grouping centers. In the case of few grouping centers, the ability to recognize visual attributes will be weakened due to too many collecting centers, the generalization is reduced, and noise is easily added, which also leads to higher computer costs.
3. The histogram of the image shows that all feature descriptors of the image are divided into groups with the closest distance from the center. After separating all attribute IDs, count the number of described attributes in each group, then assign the histogram corresponding to each image.

The expression of emotional information in the speech signal is closely related to the prosodic feature information and tone quality feature information contained in the speech signal. The prosodic features of speech refer to the tone and stress of the speaker's pronunciation (Jacob, 2017).

The amplitude of the speech signal is closely related to the expression of various emotions, which can be felt immediately in life. For example, people say more when they are angry and less when they are sad. It can be seen that the amplitude property is a specific parameter required for language sensory recognition. Amplitude characterizes the energy of speech signals of different emotions. Pronunciation duration is mainly used to indicate the change of the speaker's pronunciation time in different emotional states (Régis et al., 2017).

Speech pitch is mainly used to represent pronunciation differences between speakers due to differences in audio paths. The formant is an important parameter representing the characteristics of the vocal tract. The vocal cords produce sounds with short periods of time, high responsivity, and many frequency elements. In addition to the fundamental frequency, the vibration of the audio produces a double frame. The fundamental frequency and frequency of the sound depend primarily on the strength of the lungs and the inclination of the vocal cords. When these complex sounds resonate in the mouth, some frequencies increase while others disappear. When the sound path is regarded as a sounding interval, the sound path will oscillate to a larger amplitude, forming resonance. In this case, this natural frequency is called a formant. The bandwidth energy distribution parameter is mainly used to describe the power distribution concentration of each subset of the total audio transmission (Jain et al., 2018). The speech energy distribution of speakers in different emotional states varies within each small range. Harmony ratio is mainly used to reflect the pitch of the speakers. The extracted original speech feature data is not subjected to any dimensionality reduction processing, and the emotion recognition experiment is directly carried out. The results are shown in Table 3.

**Table 3:** Speech emotion recognition results without dimensionality reduction.

**Emotion type**	**Anger**	**Happy**	**Sad**	**Neutral**
Anger	165	25	8	2
Happy	37	135	23	5
Sad	18	36	125	21
Neutral	2	18	14	166

**Figure 5:**
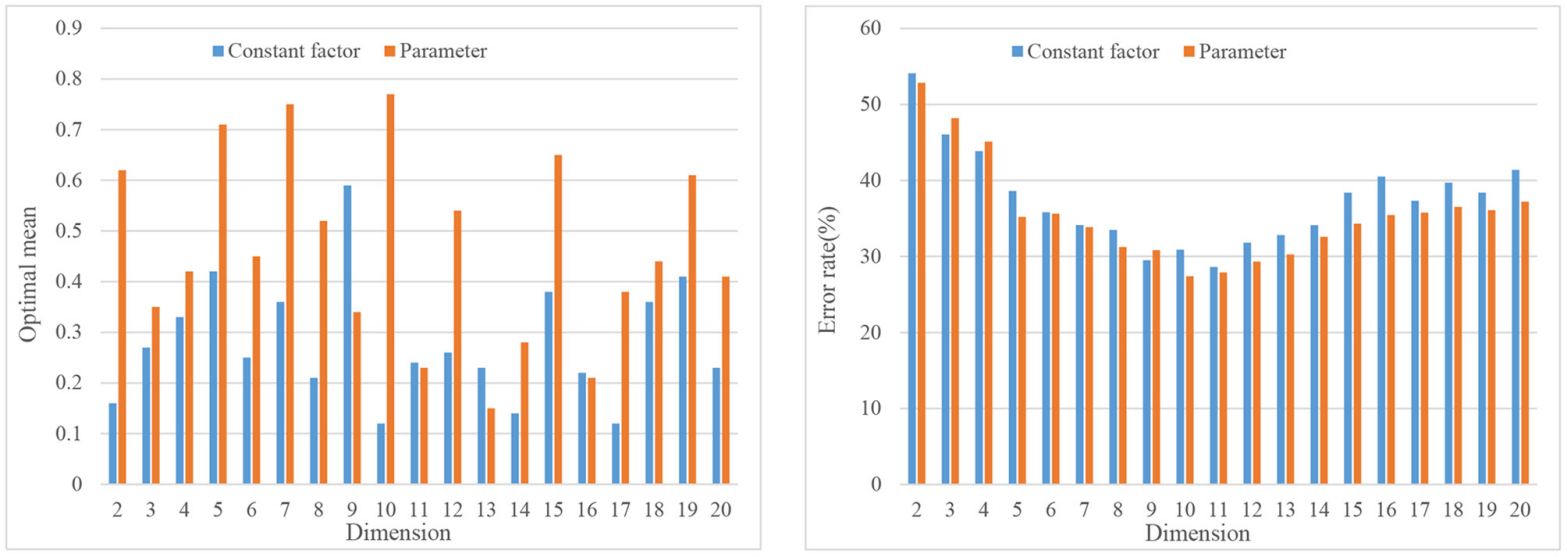
Optimizing mean and error rate.

Appropriate normalization algorithm is used to reduce the initial speech data features, then emotion recognition is performed on the low-dimensional data features, and the recognition results are compared (Wasser et al., 2018). The test results are shown in Figure 5.

**Figure 6:**
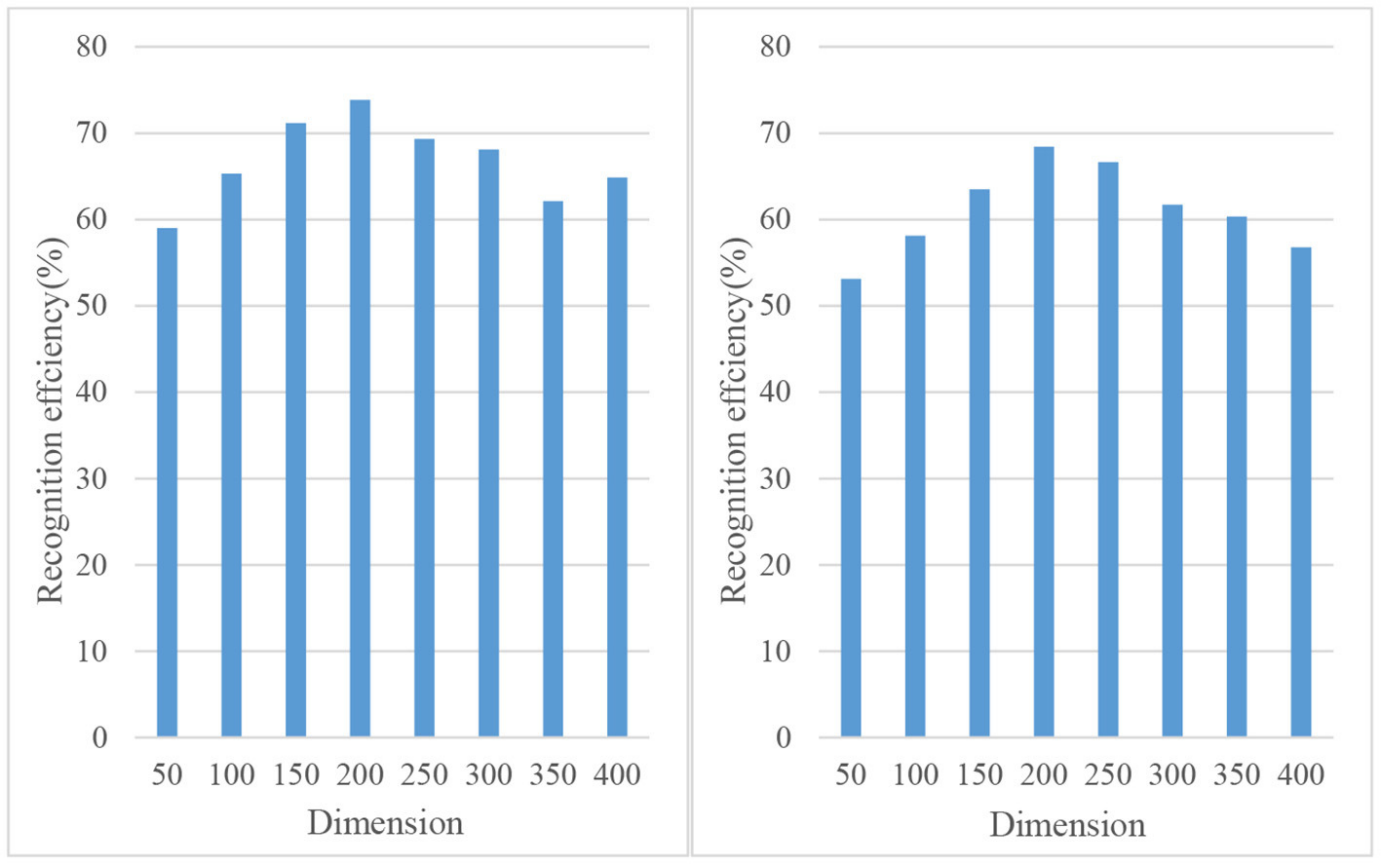
Experimental results of different dimensions and different normalization methods.

The features of the local area or key points of the expression are extracted, and then the classifier is used to classify and identify the extracted expression features (Liu et al., 2018). Using two normalization methods, the experimental results are shown in Figure 6.

**Figure 7:**
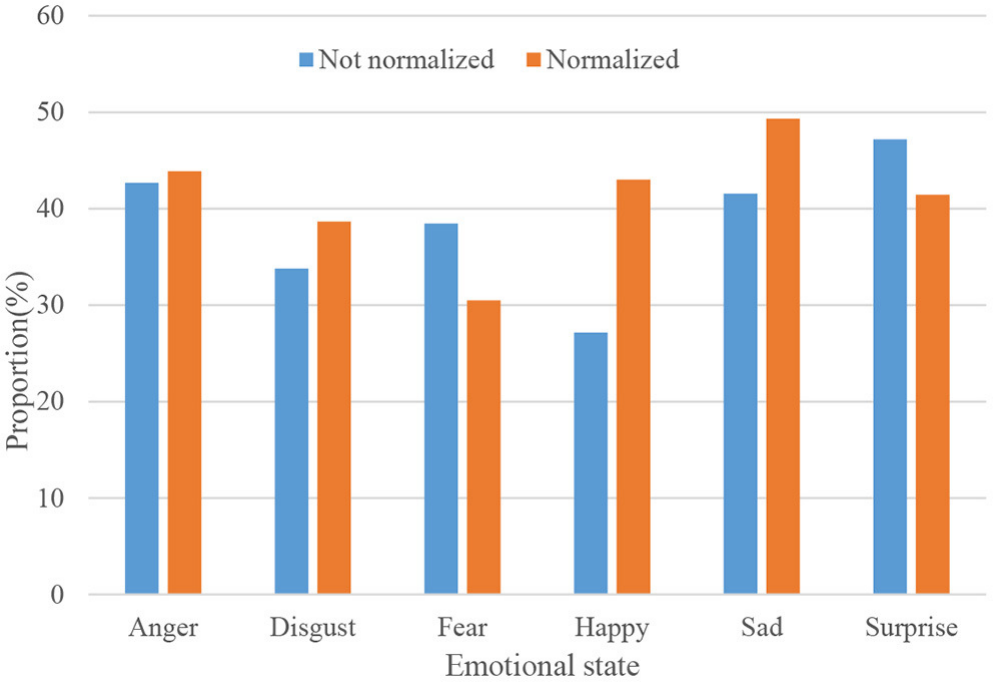
Speech emotion recognition rate under normalized and unnormalized conditions.

Figure 7 shows the normalized and unnormalized speech emotion recognition rates.

The weighting coefficient ω in the weighted summation rule of multimodal decision layer fusion can be calculated by the following Formula:


(21) $$\begin{array}{l} \frac{\omega_{m}}{\omega_{n}}=\frac{Accuracy_{m}}{Accuracy_{n}},\omega_{m}+\omega_{n}=1 \end{array}$$

*Accuracy*_*m*_-Expression single-modal emotion recognition rate

*Accuracy*_*m*_-Speech single-modal emotion recognition rate

**Figure 8:**
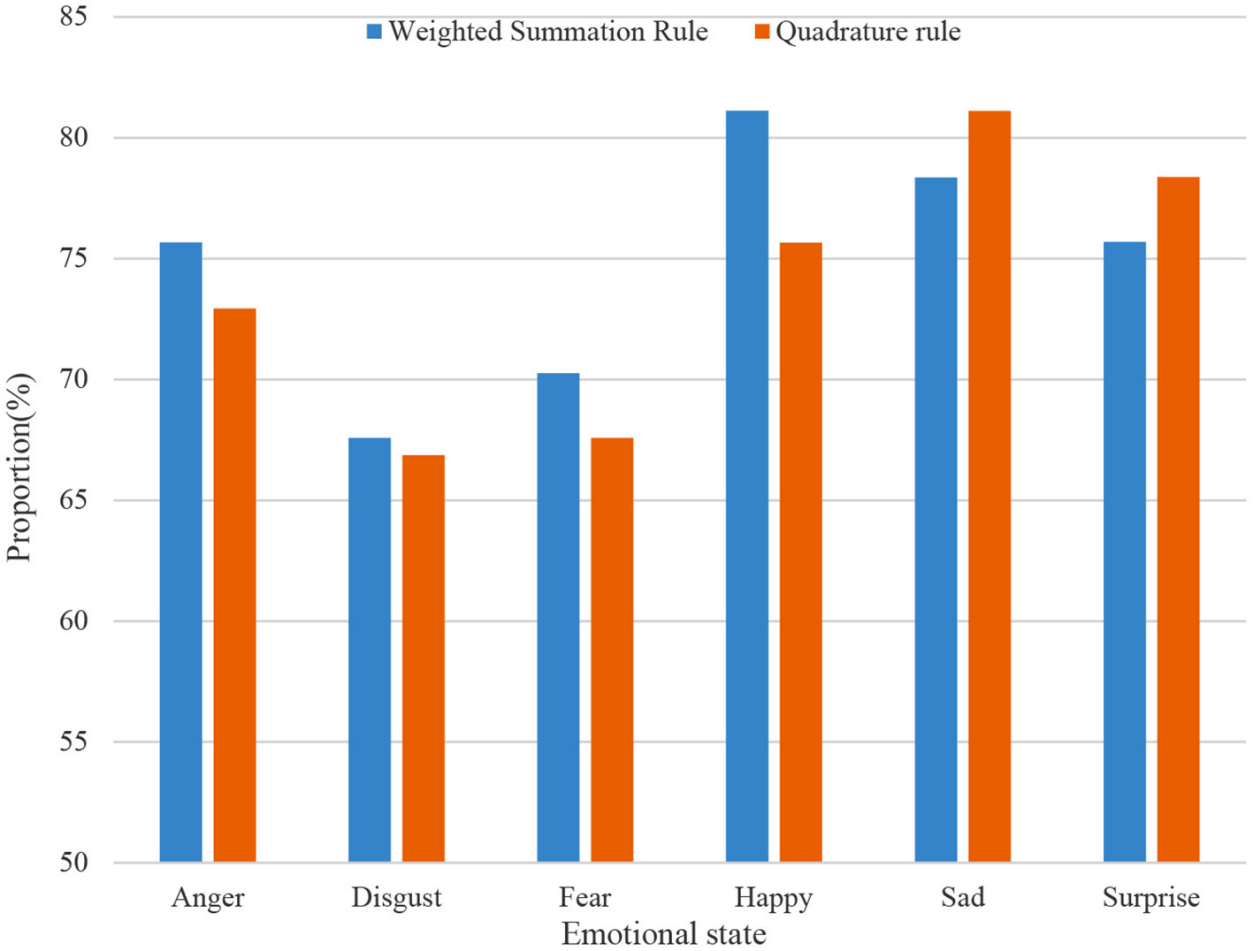
Based bimodal emotion recognition on decision layer fusion.

The experimental results of the fusion of decision layers are shown in Figure 8.

From the result data in the figure, it can be seen that the decision-making layer fusion is still very effective in bimodal emotion recognition. In general, the weighted summation rule performs slightly better than the quadrature rule in decision-making fusion, and the recognition rate reaches 76.58%. In addition, both the happiness in the summation rule and the sadness in the quadrature rule are correctly identified over 80 percent of the time. It can be intuitively found that the recognition rate of each emotional state after the fusion of the decision-making layer has narrowed the large difference shown in the single-modal emotion recognition.

**Figure 9:**
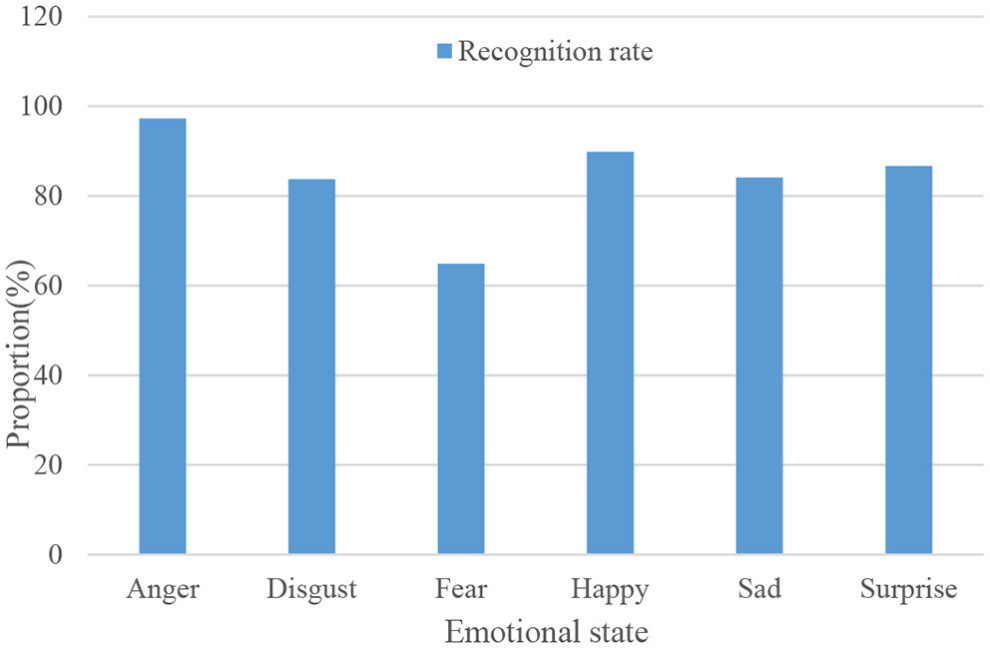
Multimodal emotion recognition success rate.

One thousand audios of anger, disgust, fear, happiness, sadness, and surprise were taken for emotion recognition, and the recognition results are shown in Figure 9.

In today's educational philosophy, we advocate independent learning. However, the status of teachers cannot be ignored. Teachers still play an important role, but the function of teachers has changed. Teachers have evolved from instilling knowledge to students into directors and organizers in teaching. Emotional factors can have a positive or negative impact on learning outcomes. Teachers and students need to pay more attention to the impact of emotional factors on learning outcomes. Teachers should grasp and understand the emotional state of students in time, and more importantly, they should pay attention to and observe the negative emotional factors of students in the learning process. Teachers should eliminate the negative influence of negative emotional factors on positive emotions, so that these factors should help to shape students and make learning in a relaxed and pleasant atmosphere. Emotional factors play an important role in the learning process: emotional factors directly and indirectly affect students' learning to varying degrees. Therefore, teachers should avoid the negative effects of emotional factors and make full use of the positive effects of emotional factors. At the same time, students should recognize the negative impact of emotional factors on the learning effect, and strive to learn to control and alleviate their emotions to reduce the negative impact (Sidera et al., 2017).

Whether it is a short-term situation or a relatively long-term stable state, positive emotions have a certain strong impact on human perception. Compared with neutral and negative emotional states, positive emotions increased the expected sequence of actions and the range of human attention, and increased creativity associated with new experiences. Positive emotions can improve problem-solving efficiency and flexibility of personal thinking. The impact of positive emotions on students' learning strategies is mainly reflected in metacognitive strategies. Positive emotions can allow students to participate in the pre-work and learning process and maintain a good emotional state. Asking learning-related questions during the learning process, and develop an effective learning plan that matches actual situation. During the study, monitor and control their own learning process, and correct their own learning behavior; after the study is over, summarize their learning results. The influence of positive emotions on students' learning strategies is mainly reflected in cognitive strategies, according to which positive emotions help students to better memorize learning information. Linking existing knowledge with new knowledge improves understanding of new knowledge, and new problems can be constructed using previous knowledge in learning (Fahad et al., 2021; Li et al., 2021; Liu and Fu, 2021).

In terms of the connotation of learning strategies, there are mainly the following viewpoints. First, learning strategies are a general term for various methods. Learning strategy is the sum of various methods of acquiring knowledge in a specific learning situation. Second, learning strategies are ways of learning regulation. Some scholars believe that learning strategies are learners' understanding of learning tasks, the use of learning methods and the monitoring of learning processes in the learning process.

A group meeting to guide students' positive emotions was organized, a control group and an experimental group were created, and there was no significant difference in the positive emotions of the experimental group and the control group before the group meeting. The test results are shown in Table 4.

**Table 4:** Positive sentiment survey data.

**Group**		**Hardly**	**A bit less (%)**	**Intermediate** **(%)**	**More (%)**	**Much**
Test group	Before counseling	0	55.14	40.78	4.08	0
	After counseling	0	6.08	34.73	46.94	12.25
Control group	Before counseling	0	32.61	34.74	20.45	12.2
	After counseling	0	6.04	40.9	44.9	8.16

**Figure 10:**
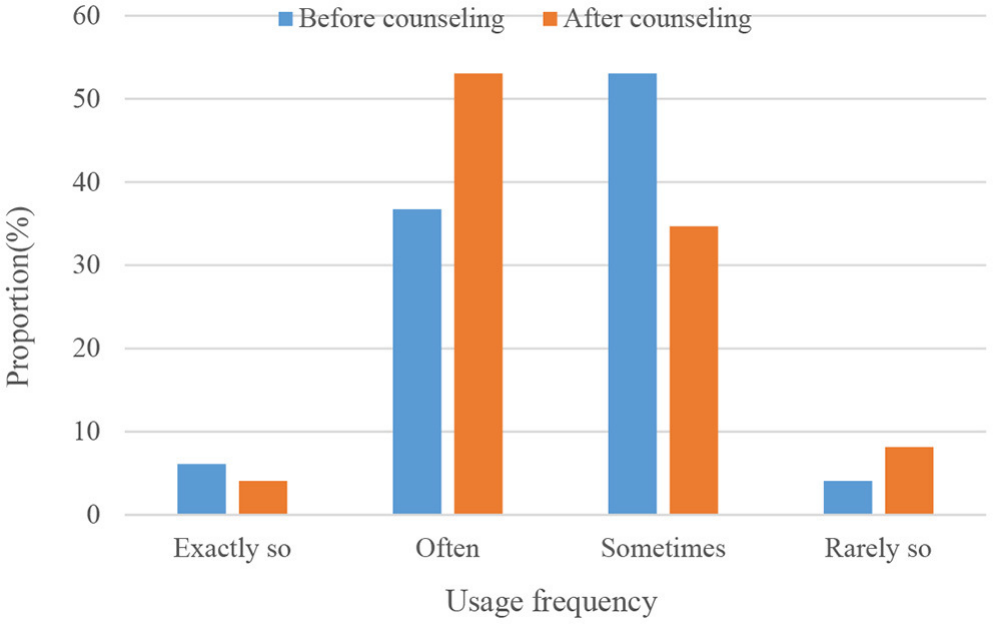
Students' use of learning strategies.

As shown in Figure 10, students use learning strategies before and after positive emotion group coaching.

To sum up, the use of emotion recognition can effectively detect the emotional state of students. For students with negative emotions, teachers should conduct group counseling to stimulate their positive emotions, so that they can learn more effectively and achieve adaptive higher education management.

## Discussion

In the construction of higher education management system, it is necessary to adhere to the construction of network, and build a good communication channel between students, teachers and administrators. Teaching management deals with the relationship between teaching individuals, and the relationship between individuals can be abstracted from the data level. Its core lies in data management, and flexible use of network technology and database technology is the best way to deal with this management problem. At the same time, the Formulation of the system is also an important guarantee for the normal operation of teaching management. The educational administration system is designed to solve the data problems in educational administration, and its purpose is to solve various problems in the flow of educational data (Farouk et al., 2015; Abulkasim et al., 2019; Hwang et al., 2020; Adil et al., 2021).

As the most intuitive way of expressing emotions in the process of human communication, facial expressions play a huge role in emotional communication. The complexity and variety of expressions often confuse people and may obscure what people really think. When human beings express emotional information, there are often not only one expression method, and even to some extent, various expression methods also have certain complementary functions when expressing emotional information. For example, when humans are happy, the rhythm of speech is usually more cheerful, which is reflected in the tone and speed of speech. At the same time, some facial muscle movements such as smiling and squinting will also appear on the human face. At this time, language and expressions also convey a pleasant emotional state. However, when a person is sad, he usually does not speak much, so the single-modal information of language cannot be used for emotion recognition, and the sad expression is usually accompanied by drooping face and mouth corners, frowning, etc. At this time, language and facial expressions complement each other in expressing emotional information. Therefore, it is more in line with the natural expression of human behavior to recognize emotion in two ways that combine facial expressions and language, rather than recognizing one-way emotion of facial expression and recognizing emotion of one mode of operation.

There are four types of unique expression feature extraction: obtaining deformation features, obtaining statistical features, distinguishing motion characteristics, and distinguishing model signals. Among them, the method of distinguishing deformation features is to obtain specific information of deformed faces, such as texture changes or geometric deformations. Methods of distinguishing statistical features include using statistical methods to describe attributes of facial expressions. One way to distinguish motion characteristics is to obtain motion information of a specific feature area or feature point, such as the change of the photocurrent in the feature area or the moving distance of the feature point. The process of distinguishing model features includes creating a 2D or 3D model based on the shape and structure information of the face in the face image, and then adjusting the changes in model parameters according to different parts of the model. Finally, the parameters of these models are regarded as the extracted model features for facial expression recognition.

The daily teaching management of colleges and universities in the teaching operation process includes the following: course management, examination management, classroom management, student status management, grade management, textbook management, official document management, teaching supervision, student reward and punishment management, teacher reward and punishment management, teacher teaching workload accounting management, file management, etc. And all of this is inseparable from data management.

## Conclusion

Emotion recognition in the field of human-computer interaction is to use computers to analyze and process speech signals, visual cues and physiological signals to determine a person's emotional state, monitor people's emotions or thought behavior, and the physical interaction between them. People mainly express their emotions through language, facial expressions, and body movements. The information of language and facial expressions are the two main indicators of human emotion. In this paper, the recognition of facial expressions and voices is carried out, and then the research on adaptive higher education management is carried out. After emotion recognition of college students, positive emotion guidance is provided for students with negative emotions. In this paper, the preliminary prediction research is carried out. In view of the limited data sources and academic level, there are inevitably some omissions in the research. The analysis of the status quo analysis stage of this paper is not thorough enough, it only shows the changes of relevant indicators, and lacks internal judgment analysis. In the stage of theoretical research, this thesis does not grasp the theory deeply enough. Emotion itself is a very complex psychological and physiological phenomenon, and its expressions are not limited to facial expressions and voice, such as body posture, physiological signals (brain, electromyography, and electrocardiogram). Therefore, in order to more accurately grasp, analyze and identify the emotional state of people, it is necessary to integrate more modal features for research. Deep learning is the current research trend in the field of machine learning and artificial intelligence. It can automatically learn the features of sounds, images or other patterns by building a deep network. Many experiments have been done in single-modal emotion recognition of facial expressions and speech, and the experimental results show that this method can achieve very good recognition accuracy.

## Data Availability Statement

The original contributions presented in the study are included in the article/supplementary material, further inquiries can be directed to the corresponding author/s.

## Author Contributions

HZ: writing and editing. ZL: data analysis. All authors contributed to the article and approved the submitted version.

## Funding

This work was supported by the projects of the Human Social Science on Young Fund of the Ministry of Education Research on the Institutional History of French Communication under the New Cultural History Paradigm (19YJC860031).

## Conflict of Interest

The authors declare that the research was conducted in the absence of any commercial or financial relationships that could be construed as a potential conflict of interest.

## Publisher's Note

All claims expressed in this article are solely those of the authors and do not necessarily represent those of their affiliated organizations, or those of the publisher, the editors and the reviewers. Any product that may be evaluated in this article, or claim that may be made by its manufacturer, is not guaranteed or endorsed by the publisher.
